# Umbilical Hernia in a Ram and Its Successful Surgical Repair

**DOI:** 10.1002/ccr3.73621

**Published:** 2026-09-28

**Authors:** Getachew Tadesse Siyoum

**Affiliations:** ^1^ Department of Veterinary Medicine, College of Agricultural Sciences Woldia University Woldia Ethiopia

**Keywords:** cruciate suture pattern, herniorrhaphy, ram, silk, umbilical hernia

## Abstract

This case demonstrates the successful application of a cruciate suture pattern using silk suture for umbilical hernia repair in a ram. A one‐year‐old local breed ram was presented to the Professor Feseha Gebreab Memorial Veterinary Teaching Hospital, Addis Ababa University, Ethiopia, with a progressively enlarging umbilical swelling. Clinical examination revealed a non‐painful, large umbilical hernia, for which the ram was admitted for surgical repair. Herniorrhaphy was conducted after circular infiltration of 2% lidocaine. The hernial sac was opened through a longitudinal incision cranial to the prepuce, and the herniated omentum and intestine were reduced. The hernial ring was closed using a cruciate suture pattern with size 2 silk. Postoperatively, Penicillin–streptomycin and meloxicam were administered for three consecutive days. Recovery was uneventful, with complete healing. The outcome suggests this technique is a viable and effective suture pattern for hernias of similar size, in addition to timely diagnosis, accurate surgical execution, and diligent postoperative care.

## Key Clinical Message

This case highlights the successful herniorrhaphy of an umbilical hernia in a sheep using cruciate suture pattern with size 2 silk, demonstrating this technique as a viable and effective practical alternative when combined with timely surgical intervention and diligent postoperative care.

## Graphical abstract

Description: Clinical presentation showing a distinct umbilical swelling (A). Prepared surgical site after clipping and aseptic scrubbing (B). Draped surgical field, secured with towel clamps (C). Extension of the stab incision hernia sac under digital guidance (D). Hernial ring after reduction of the herniated contents (E). Closure of the hernial ring by herniorrhaphy (F). Immediate postoperative appearance of the ram in a standing position (G). Follow‐up at 60 days postoperatively, demonstrating complete recovery, excellent health, and body weight gain (H).

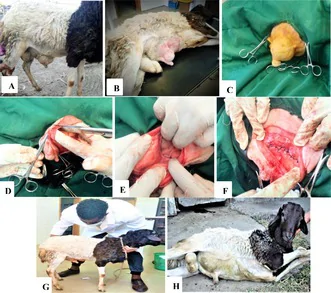

## 1 Introduction

Hernia is the abnormal protrusion of tissue or organs through a defect or natural opening within intact skin. Its anatomical structure consists of three components: the sac (the swelling itself), its contents, and the ring (the opening, which may be natural or acquired) [1]. Based on location, hernias are classified into several types, including scrotal, inguinal, abdominal, diaphragmatic, perianal, and umbilical [2].

An umbilical hernia is characterized by the protrusion of abdominal contents through the umbilical ring, resulting from incomplete closure of the abdominal wall at the navel. The opening of the umbilicus closes within a few days after birth; however, failure to close may be due to congenital or acquired factors [3]. Congenital umbilical hernias are present at birth and often have a hereditary component. In contrast, acquired hernias manifest several weeks postnatal, with predisposing factors including infection, trauma, and increased intra‐abdominal pressure [4, 5].

The clinical presentation of umbilical hernias ranges from minor to severe. Most are small, reducible, and clinically silent. However, larger hernias may become irreducible, posing a risk of incarceration and subsequent strangulation, which can present with acute, severe clinical signs [4]. The diagnosis of a hernia relies on the patient's history, physical examination, and diagnostic imaging [6]. The specific diagnostic approach varies by hernia type. For umbilical hernias, a visible swelling at the umbilicus is common. Diagnosis is often confirmed by manually reducing the herniated mass and palpating the hernia ring, a procedure typically facilitated by positioning the animal in dorsal recumbency [7].

Umbilical hernia must be differentiated from other common umbilical swellings and abnormalities, such as umbilical cysts, hematomas, tumors, and abscesses. An umbilical abscess is typically distinguished by local inflammatory signs; it may not fluctuate readily under the skin and often shows a tendency to point or discharge purulent material. A hematoma in the umbilical region, resulting from vascular trauma, feels like free fluid and may produce subtle crepitus on palpation and bloody content during aspiration. An umbilical cyst fluctuates uniformly, lacks pain or functional symptoms, and shows no tendency to point. A definitive diagnosis of an umbilical hernia relies on the pathognomonic identification of a palpable umbilical ring, a hernial sac, and reducible contents. Supplementary diagnostic measures, including ultrasonography, radiography, or abdominocentesis, can provide further confirmation when reducibility is unclear due to adhesions or swelling [7, 8].

The management of an umbilical hernia can be either surgical or non‐surgical, with the choice of treatment determined by factors such as the size of the hernia ring, the duration of the condition, and the overall clinical severity [6]. Non‐surgical interventions typically include the use of supportive bandages or specialized hernia clamps. In contrast, surgical correction via herniorrhaphy or hernioplasty is the standard approach for large rings or cases complicated by infection [6, 9]. Therefore, this case report aimed to describe and document the clinical presentation and successful surgical management, specifically herniorrhaphy, of an umbilical hernia in a local breed of ram.

## 2 Case Description and Its Managements

### 2.1 History of the Case

A one‐year‐old 21 kg local breed ram was presented to the Professor Feseha Gebreab Memorial Veterinary Teaching Hospital at the College of Veterinary Medicine and Agriculture, Addis Ababa University, with the complaint of a one‐month history of a progressively enlarging umbilical swelling. The owner reported no history of trauma and previous treatment. The ram was managed semi‐intensively within a flock and had a normal feed intake.

### 2.2 Clinical Examination

Upon clinical examination, the ram was alert, with vital parameters within normal physiological ranges: heart rate of 74 beats per minute, respiratory rate of 24 breaths per minute and rectal temperature of 39.0°C. Physical inspection of the umbilical region revealed a localized, spherical swelling (Figure 1A). Palpation confirmed the presence of a distinct hernial ring, a hernial sac, and soft, non‐painful contents. When the ram was placed on dorsal recumbency, the contents were fully and manually reducible into the abdominal cavity under gentle pressure. Exploratory abdominocentesis at the swelling of umbilicus yielded no fluid or purulent discharge, ruling out localized abscess or hematoma. Based on the history and physical examination, the condition was diagnosed as an umbilical hernia, and surgical repair (herniorrhaphy) was recommended.

**FIGURE 1:**
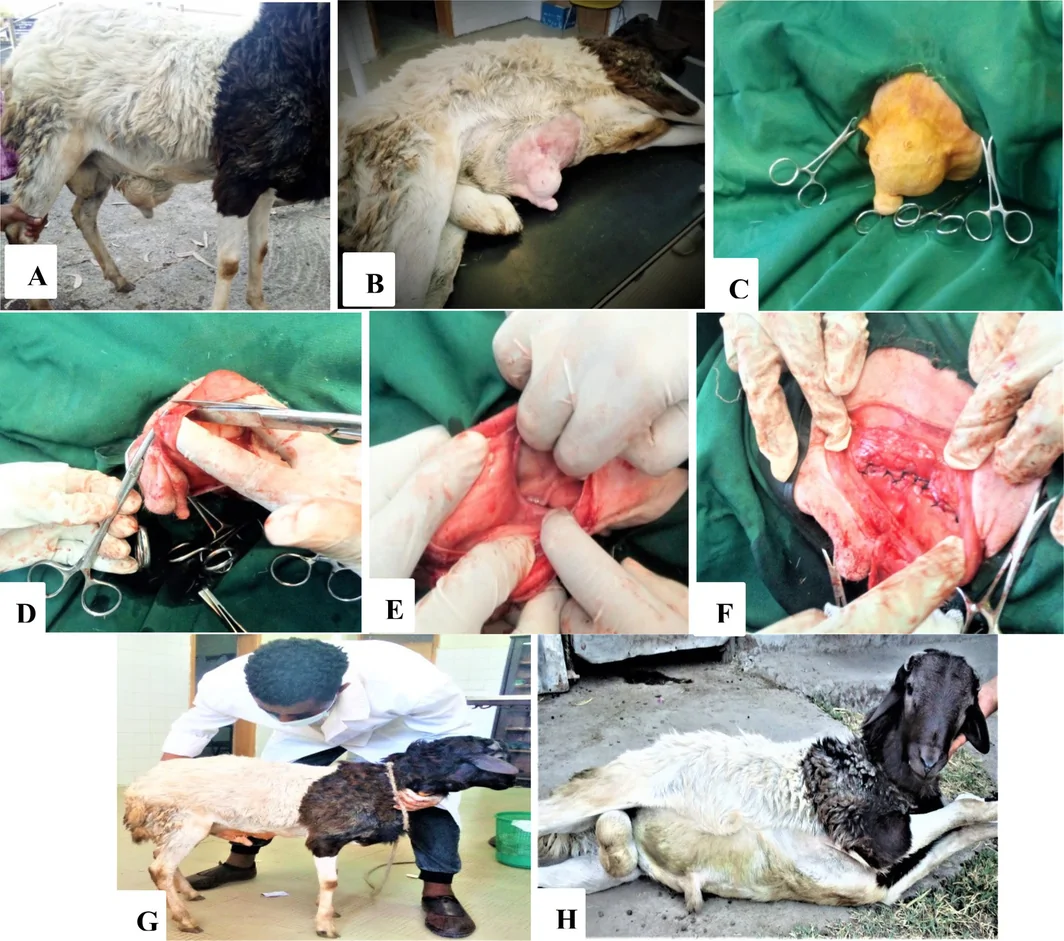
Clinical presentation, surgical repair, and outcome of an umbilical hernia in a ram. Clinical presentation showing a distinct umbilical swelling. (A) Prepared surgical site after clipping and aseptic scrubbing. (B) Draped surgical field, secured with towel clamps. (C) Extension of the stab incision of hernial sac under digital guidance (D) Hernial ring after reduction of the herniated contents. (E) Closure of the hernial ring by herniorrhaphy. (F) Immediate postoperative appearance of the ram in a standing position (G). Follow‐up at 60 days postoperatively, demonstrating complete recovery, excellent health, and body weight gain (H).

### 2.3 Preoperative Preparation

The owner was instructed to re‐present the ram the following day after an overnight fast from feed and water withheld for 12 h. Upon presentation for the scheduled procedure, the vital parameters were evaluated; all were within normal physiological range of values. The umbilical swelling and surrounding region were initially washed with soap and water. The site was then closely shaved, scrubbed and thoroughly cleansed with water and an antiseptic solution containing cetrimide and chlorhexidine gluconate (Savlon Antiseptic Liquid, ITC Limited, Kolkata, India) (Figure 1B). Surgical preparation was completed by alternating the antiseptic scrub with 70% isopropyl alcohol. Following this, the surgical field was painted with a 2% iodine tincture in a centrifugal fashion (concentric circles moving outward from the intended incision site). Finally, the prepared area was draped using a sterile, fenestrated surgical drape, secured with towel clamps (Figure 1C).

### 2.4 Anesthesia and Animal Control

The ram was placed and secured in dorsolateral recumbency on the surgical table by humanely tying the fore and hind limbs separately using soft ropes. Local infiltration anesthesia was achieved using 2% lidocaine hydrochloride (Mackur Laboratories Ltd., Gujarat, India). The anesthetic agent was administered circumferentially around the base of the hernia at a total dose of 4 mg/kg body weight (infiltrated at approximately 0.5–1.0 mL per 1–2 cm around the swelling).

### 2.5 Surgical Site and Technique

The proposed incision site was directly over the apex of the hernial swelling, and herniorrhaphy was performed.

### 2.6 Surgical Procedures and Corrections

A single longitudinal skin incision was made directly over the swelling in the midline, anterior to the prepuce. This incision was extended as needed by curving it laterally to the right of the prepuce. Hemostasis was maintained throughout the procedure using direct gauze pressure and hemostatic artery forceps. The deeper subcutaneous tissues and underlying fascia were carefully dissected using a blunt technique. The deeper subcutaneous tissues and underlying fascia were carefully dissected using a blunt technique with blunt closed Mayo scissor, allowing the hernial sac to be completely separated from the surrounding tissues. The hernial sac was then grasped with tissue forceps and tented upward away from the underlying viscera. A small stab incision was made into the elevated sac using a #11 scalpel blade. The index finger was inserted through the stab incision to palpate and clear any adhesions between the sac and the hernial contents before extending the incision to fully expose the hernial contents and hernial ring.

The hernia sac was then fully opened by extending the initial stab incision with Mayo scissors (Figure 1D). Upon opening the sac, the herniated contents, comprising the omentum and intestine, were identified and carefully reduced back into the abdominal cavity. Following reduction, a circular hernia ring measuring approximately 2–3 cm in diameter was clearly visualized (Figure 1E). The hernia sac was retracted laterally using hand‐held retractors to expose the hernial ring. The fibrotic edge of the hernial ring was refreshed using a scalpel blade to create a clean, viable surface for healing. To facilitate a tension‐free appositional closure, the ring was enlarged by making two small, sharp relief incisions, each approximately 1 cm long, in opposite directions cranially and caudally.

The hernial ring was closed using a cruciate suture pattern with size 2 silk suture (Figure 1F). Closure was achieved by placing all sutures first and then tightening them sequentially in a centrifugal center‐to‐peripheral fashion, progressing from the center of the defect toward its lateral margins. Following ring closure, procaine penicillin powder was applied topically over the sutured fascial site for local antimicrobial prophylaxis. The excess hernial sac, redundant skin, and subcutaneous tissue was trimmed away. To obliterate dead space and withstand postoperative abdominal wall tension, the subcutaneous layer was closed with a simple continuous suture pattern using size 2 polyglactin 910 (Vicryl). Skin apposition was achieved with a horizontal mattress pattern using size 2 silk to ensure robust mechanical security against animal movement. Finally, the surgical site was dressed with a 2% iodine solution. The surgery was completed successfully (Figure 1G), and the ram was discharged.

### 2.7 Postoperative Care and Outcome

Postoperative care consisted; wound care and a three‐day antimicrobial and analgesic regimen. A combination of procaine penicillin G and dihydrostreptomycin sulfate (Penstrep‐400, Interchemie Werken De Adelaar B.V., Venray, Netherlands) was administered intramuscularly at a dose of 0.1 mL/kg body weight, once daily for three consecutive days for infection prophylaxis. Concurrently, meloxicam (Meloxicash, Ashish Life Science Pvt. Ltd., Maharashtra, India) was administered intramuscularly at 0.3 mg/kg body weight once daily for three consecutive days for analgesia.

The surgical wound was cleansed and re‐dressed with 2% iodine tincture every other day until complete healing was achieved. Skin sutures were removed on the 15th postoperative day. The ram recovered fully, and the surgical site healed without complications. During follow‐up examinations conducted over a two‐month period at 30 and 60 days postoperatively, the ram remained in good health, gained weight, and showed no signs of recurrence (Figure 1H).

## 3 Discussion

Hernias can significantly reduce reproductive and productive performance in animals [10]. Umbilical hernias may be congenital, occurring at or shortly after birth, or acquired later in life due to factors such as trauma, infection, or increased intra‐abdominal pressure [11]. The present case of umbilical hernia in a one‐year‐old ram was considered to be an acquired hernia of unknown etiology. This aligns with a previous study [2] that reported acquired umbilical hernias in sheep ranging from four months to four years of age.

In the present case, local anesthesia was achieved via circular infiltration of 2% lidocaine surrounding the base of the hernia, without supplemental systemic sedation or general anesthesia, to eliminate the risks of bloat, regurgitation, aspiration pneumonia, and prolonged cardiopulmonary depression. This technique is consistent with previous reports describing circular field infiltration for umbilical hernia repair in small ruminants [2]. However, local infiltration of 2% lidocaine combined with sedation using 2% xylazine has also been reported for herniorrhaphy in sheep [5]. The selection of an anesthetic protocol depends on patient temperament, hernia complexity, animal stability, and resource availability. Regarding the surgical approach, the skin incision for umbilical hernia repair in male small ruminants is routinely performed cranial or lateral paramedian to the prepuce [5, 9], to avoid inadvertent injury to the preputial sheath and penile structures, which is similar to the approach used in the current case.

In veterinary practice, various herniorrhaphy suturing techniques are employed for umbilical hernia repair [12]. Commonly reported methods include the use of interrupted and overlapping horizontal mattress pattern with non‐absorbable suture material, as described in several studies [2, 5, 12, 13]. These studies further detailed techniques involving interrupted horizontal mattress patterns, supplemented by an additional row of simple interrupted sutures using nylon size 2. However, in the present case, herniorrhaphy was performed using a cruciate suture pattern with size 2 silk, after which the ram was healed successfully.

## 4 Conclusion

This case report describes and documents the successful surgical management of an umbilical hernia in a one‐year‐old ram using herniorrhaphy under local anesthesia, employing a cruciate suture pattern with size 2 silk. The ram recovered successfully without complications, demonstrating the efficacy and viability of this surgical technique as a practical alternative to the more commonly reported horizontal mattress patterns. Based on the outcome, the cruciate suture pattern is recommended as an effective technique for repair of reducible umbilical hernias of similar size in sheep. For optimal results, surgical intervention should be supported by timely diagnosis, accurate surgical execution, and diligent postoperative care, including appropriate antimicrobial and analgesic protocols, to ensure successful healing and prevent complications. As a limitation of this case report, larger prospective clinical studies involving a broader case series are required to further validate the long‐term outcomes and comparative efficacy of this technique.

## Author Contributions

**Getachew Tadesse Siyoum:** conceptualization, methodology, investigation, data curation, software, resources, validation, supervision, writing – original draft, writing – review and editing, visualization.

## Funding

The author has nothing to report.

## Ethics Statement

In accordance with the institutional policies of Addis Ababa University, College of Veterinary Medicine and Agriculture, formal approval from an animal ethics committee was not required for a case report.

## Consent

Informed verbal and written consent was obtained from the animal's owner for the procedure and for the use of clinical data and images for scientific publication.

## Conflicts of Interest

The author declares no conflicts of interest.

## Data Availability

Data sharing not applicable to this article as no datasets were generated or analysed during the current study.
